# Fibroblast-derived CXCL12 increases vascular permeability in a 3-D microfluidic model independent of extracellular matrix contractility

**DOI:** 10.3389/fbioe.2022.888431

**Published:** 2022-09-02

**Authors:** Jacob C. Holter, Chia-Wen Chang, Alex Avendano, Ayush A. Garg, Ajeet K. Verma, Manish Charan, Dinesh K. Ahirwar, Ramesh K. Ganju, Jonathan W. Song

**Affiliations:** ^1^ Department of Biomedical Engineering, The Ohio State University, Columbus, OH, United States; ^2^ Department of Chemical and Biomolecular Engineering, The Ohio State University, Columbus, OH, United States; ^3^ Department of Mechanical and Aerospace Engineering, The Ohio State University, Columbus, OH, United States; ^4^ Department of Pathology, College of Medicine, The Ohio State University, Columbus, OH, United States; ^5^ Comprehensive Cancer Center, The Ohio State University, Columbus, OH, United States; ^6^ Department of Bioscience and Bioengineering, Indian Institute of Technology, Jodhpur, RJ, India

**Keywords:** microfluidics, tumor microenvironment, cancer-associated fibroblast, paracrine signaling, collagen hydrogel, microtissue analogue

## Abstract

Cancer-associated fibroblasts (CAFs) play an active role in remodeling the local tumor stroma to support tumor initiation, growth, invasion, metastasis, and therapeutic resistance. The CAF-secreted chemokine, CXCL12, has been directly implicated in the tumorigenic progression of carcinomas, including breast cancer. Using a 3-D *in vitro* microfluidic-based microtissue model, we demonstrate that stromal CXCL12 secreted by CAFs has a potent effect on increasing the vascular permeability of local blood microvessel analogues through paracrine signaling. Moreover, genetic deletion of fibroblast-specific CXCL12 significantly reduced vessel permeability compared to CXCL12 secreting CAFs within the recapitulated tumor microenvironment (TME). We suspected that fibroblast-mediated extracellular matrix (ECM) remodeling and contraction indirectly accounted for this change in vessel permeability. To this end, we investigated the autocrine effects of CXCL12 on fibroblast contractility and determined that antagonistic blocking of CXCL12 did not have a substantial effect on ECM contraction. Our findings indicate that fibroblast-secreted CXCL12 has a significant role in promoting a leakier endothelium hospitable to angiogenesis and tumor cell intravasation; however, autocrine CXCL12 is not the primary upstream trigger of CAF contractility.

## Introduction

The emerging role of the tumor microenvironment (TME) on cancer development has become increasingly evident over the past two decades, highlighted within the hallmarks of cancer (Hanahan, 2022). Along with cancer cells, the host of nonmalignant cellular and noncellular components that arise during the course of tumorigenesis results in a local tumor niche, or TME (Valkenburg et al., 2018). These non-cancer-cell constituents of the tumor stroma include cancer-associated fibroblasts (CAFs), immune cells, endothelial cells (ECs), pericytes, and adipocytes, as well as extracellular matrix (ECM) components, among others (Valkenburg et al., 2018; Jin and Jin, 2020). Increasing evidence shows that a set of these cell types are co-opted by cancer cells and cancer stem cells (CSCs) contributing to tumor initiation, growth, invasion, metastasis, and therapeutic resistance (Jin and Jin, 2020). As a result, stromal cells are widely believed to contribute to the initiation of epithelial carcinomas and subsequent tumor progression (Wiseman and Werb, 2002; Orimo et al., 2005). Specific to breast cancer, clinical evidence and various neoplastic models have linked mammary stromal cells to tumorigenesis (Finak et al., 2008; Trimboli et al., 2009; Maller et al., 2021). Despite these discoveries implicating the tumor stroma in carcinogenesis, a robust understanding of the cell signaling pathways between various components of the TME remains to be seen. Therefore, elucidating the signaling that underpins stromal-epithelial crosstalk and communication amongst stromal cells is critical to progress the translation of TME-based anticancer therapies and address the broad clinical challenge of metastasis.

Fibroblasts are found in various carcinomas and are often the most abundant cell population of the tumor stroma (Hanahan and Weinberg, 2011; Kalluri, 2016). While normal fibroblasts have multiple physiological functions—from the connective tissue-producing role of secreting fibrous collagens to the recruitment of immune cells and regulation of inflammation—CAFs play an active role in reshaping the TME to support tumor cell survival and proliferation (Houthuijzen and Jonkers, 2018). In breast cancer, CAFs can originate from a variety of sources, including cancer stem cells (Nair et al., 2017) and reprogrammed native fibroblastic cells (Kojima et al., 2020); however, independent of their origin, CAFs are classified by their activated state resembling myofibroblasts during inflammation and wound healing (Shiga et al., 2015). The activated state of CAFs leads to the excessive deposition of various ECM proteins, e.g., type I collagen, thereby implicating CAFs in the creation of a desmoplastic stroma common in epithelial tumors, including breast cancer (Orimo et al., 2005). In addition to fibrosis and increased matrix stiffness (Mouw et al., 2014), CAFs have been demonstrated to induce cancer proliferation and enhance migration, invasion, and distant metastasis in breast cancer (Houthuijzen and Jonkers, 2018).

One of the more recent strategies to combat the effects of CAFs on tumorigenesis has been to target CAF-secreted growth factors (Park et al., 2014) and chemokines, e.g., stromal cell-derived factor 1 (SDF-1), also known as CXCL12 (Chen et al., 2019). The success of this approach relies heavily on a comprehensive understanding of the signaling network between CAFs and other cellular constituents of the TME. In 2005, Orimo et al. first showed that CAFs play a central role in promoting the proliferation of breast cancer cells through their ability to secrete CXCL12 (Orimo et al., 2005). CXCL12 acts directly on mammary carcinoma cells through the cognate receptor CXCR4 to stimulate tumor growth (Müller et al., 2001; Littlepage et al., 2005; Luker et al., 2012). Additionally, CAFs were able to induce angiogenesis through CXCL12-mediated recruitment of endothelial progenitor cells (EPCs) (Orimo et al., 2005). As an extension of this pioneering work, continued efforts have been made to characterize the regulatory role of fibroblast-derived CXCL12 within the TME and its mechanistic effect on metastasis (Ahirwar et al., 2018). Herein, we demonstrate in an *in vitro* microfluidic model that stromal CXCL12 secreted by CAFs enhances vascular permeability. These findings suggest that CXCL12 secreted by CAFs promotes metastasis through the expansion of a leaky tumor vasculature. Furthermore, we evaluate the autocrine role of CXCL12 on CAFs’ ability to contract and remodel the ECM. These findings collectively bolster the understanding of fibroblast-derived CXCL12 to facilitate tumor angiogenesis and intravasation, ultimately leading to metastasis.

## Materials and methods

### Ethics statement

Usage of mice and experimentation with their harvested cells was approved by the Institutional Animal Care and Use Committee (IACUC) at the Ohio State University.

### Cell culture

Mouse embryonic endothelial cells (MEECs) were a generous gift from Dr. Nam Y. Lee of the Ohio State University (OSU). MEECs were cultured in endothelial cell media with an endothelial cell supplement kit, including 5% fetal bovine serum (FBS), vascular endothelial growth factor (VEGF) and heparin (Cell Biologics). Endothelial cells were cultured in T-75 flasks in a humidified incubator at 37°C and 5% CO_2_ with media exchange every 2 days. Cell passage numbers of 5–10 were used for the study. MEECs were harvested from flasks using 0.05% Trypsin-EDTA (Invitrogen), centrifuged, and resuspended in cell media at a concentration of ∼7 × 10^6^ cells ml^−1^. MEECs were then pipetted into both lateral microchannels of the device and allowed to adhere within the cell culture incubator overnight at 37°C to obtain an endothelial monolayer. Endothelial media was exchanged daily after seeding to promote healthy cell growth. For experimental conditions with conditioned media, cells were permitted to be cultured within the device for 1 day prior to the introduction of conditioned media to stabilize the microvessel analogues and ensure treatment arms did not influence initial cell attachment.

Normal mouse mammary gland fibroblasts (MMFs) were harvested from the mammary glands of either Cxcl12 floxed control (Cxcl12^f/f^) mice to obtain CXCL12^f/f^ fibroblasts (also denoted as “f/f” fibroblasts), or from fibroblast-specific conditional knockout (Cxcl12^∆/∆^) mice to obtain CXCL12^∆/∆^ fibroblasts (also denoted as “∆/∆” fibroblasts), as previously described (Ahirwar et al., 2018). Briefly, with respect to the knockout allele, the Cxcl12 gene was flanked by LoxP sites. To delete the LoxP-flanked Cxcl12 gene, we introduced Cre recombinase by breeding Cxcl12 transgenic mice with FSP-Cre expressing mice. The Cre recombinase expression in FSP-Cre mice is driven by fibroblast-specific protein (FSP) promoter. As FSP is specifically expressed by fibroblasts, the Cxcl12 gene in double transgenic mice (Cxcl12 f/f; FSP-Cre or Cxcl12^∆/∆^) will specifically be deleted in the fibroblasts. Normal mammary glands harvested from 8-week-old FVB female mice were minced and digested with a cocktail of collagenase IV and hyaluronidase in the presence of hydrocortisone, insulin, and antibiotics penicillin and streptomycin in a 5% CO_2_ incubator for 2 h at 37°C. Digested tissue was resuspended in a medium with 10% FBS, and supernatants were subjected four more times to gravity sedimentation for 12–15 min to obtain isolated MMFs.

To acquire cancer-associated fibroblasts (CAFs), the mouse mammary tumor virus (MMTV) promoter Polyoma middle T antigen (PyT) (Jackson laboratories) was intercrossed with either Cxcl12 floxed control (Cxcl12^f/f^) mice to generate Cxcl12^f/f^; PyT mice, or with fibroblast-specific conditional knockout (Cxcl12^∆/∆^) mice to generate Cxcl12^∆/∆^; PyT mice (Ahirwar et al., 2018). The tumor-bearing mammary glands of Cxcl12^f/f^; PyT mice were used to harvest CXCL12^f/f^; PyT cancer-associated fibroblasts (also denoted as “f/f; PyT” CAFs), while the tumor-bearing mammary glands of Cxcl12^∆/∆^; PyT mice were used to harvest CXCL12^∆/∆^; PyT cancer-associated fibroblasts (also denoted as “∆/∆; PyT” CAFs). The PyT tumors of 8-week-old FVB female mice were similarly digested with the cocktail of collagenase IV and hyaluronidase for 30 min under incubation, and CAFs were isolated by gravity sedimentation, as described above. CAFs were further enriched by differential trypsinization two times. After isolation, MMFs and CAFs were maintained in DMEM/F-12 media supplemented with insulin, hydrocortisone, epidermal growth factor, and 10% FBS (Sigma Aldrich). Fibroblast media was exchanged every 2 days during culture leading up to seeding. Fibroblasts were used in experiments at low passage numbers (2–4) to minimize potential cellular changes associated with extended culture of harvested primary cells.

### Immunofluorescence

Isolated CAFs were seeded into 4-well chamber slides (Thermo Scientific) at a concentration of 1.0 × 10^5^ cells/ml. After 24 h, cells were fixed with 4% (w/v) paraformaldehyde (PFA, Sigma-Aldrich) for 15 min at room temperature (RT), and subsequently permeabilized with 0.1% Triton X-100 (Sigma-Aldrich) for 5 min at RT. After washing with 1X PBS, cells were blocked with 5% BSA in 1X PBS for 1 h at RT. Subsequently, primary antibodies were added to wells and incubated overnight at 4°C: CXCL12 antibody at 12.5 μg/ml concentration (R&D Systems, MAB350), PDGFR-α antibody at 1:1000 dilution (CST, 3174), and Nidogen-2 at 1:400 dilution (Abcam, ab14513). After aspiration and extensive washing with 1X PBS, secondary antibodies were added and incubated for 1 h at RT: AF-594 anti-rabbit and AF-488 anti-mouse (1:1000 dilution, Invitrogen). The slide was washed with 1X PBS, and the coverslip was mounted with DAPI-containing mounting medium (Vector Laboratories). The slide was stored at 4°C until imaging. Fluorescence images were acquired on a Nikon A1R confocal microscope: representative images were taken at ×40 magnification, and quantification of PDGFR-α and Nidogen-2 was performed on ×10 images. For quantification, mean pixel intensity was quantified by NIH ImageJ software and normalized by dividing by total cell count (*n* ≥ 150 cells) to obtain intensity per cell in arbitrary units (AU).

### Western blotting

Western blot analysis was performed as previously described (Anand et al., 2013; Garg et al., 2019; Charan et al., 2020). Briefly, the cells were lysed using RIPA buffer (Thermo Scientific) supplemented with proteases and phosphatase inhibitors. Total protein was estimated with Bradford protein assay, per manufacturer’s instructions (Bio-Rad). Subsequently, 40 μg of protein was loaded on 4–12% gradient SDS–polyacrylamide gel (Invitrogen) under reducing conditions, transferred to nitrocellulose membrane, and blocked with 5% non-fat dry milk (NFDM) in Tris-buffered saline with 0.1% Tween 20 (TBST). The membrane was incubated overnight with anti-CXCR4 primary antibody (Santa Cruz, sc-53534) at a 1:200 dilution, washed 3 times with TBST, and incubated for 1 h at RT with horseradish peroxidase-conjugated secondary antibody (1:10,000) in blocking buffer. Membranes were washed and developed by using an enhanced chemiluminescence (ECL) system (Thermo Scientific) and immediately exposed to autoradiography film (GeneMate). ∆/∆; PyT CAFs from Cxcl12^∆/∆^; PyT mice were unavailable for the Western blot.

### Microfluidic microtissue analogue

The microtissue analogue system, was fabricated using soft lithography of poly (dimethylsiloxane) (PDMS) as previously reported (Chang et al., 2020). This configuration enabled us to create laterally adjacent stromal and endothelial compartments to mimic the *in vivo* conditions for fibroblast and EC interactions within the tumor stroma. Briefly, the base and curing agent of PDMS were mixed at a ratio of 10:1, respectively, and poured onto a patterned silicon wafer. After curing for at least 2 h at 65°C, the PDMS layer was subsequently bonded to a glass slide by oxygen plasma treatment. Type I collagen (Corning) sourced from rat tail tendon was pipetted into the central ECM compartment of the device at a concentration of 6 mg ml^−1^ and incubated for at least 30 min at 37°C to polymerize. For experimental co-culture conditions, CAFs were added to the collagen matrix at a density of 6 × 10^3^ cells per µl prior to polymerization and pipetted into the stromal compartment of the device in a similar fashion. Lateral microchannels were coated with fibronectin (100 mg ml^−1^) at least 30 min prior to cell seeding to improve cellular attachment and biocompatibility of the device. Upon seeding, mouse embryonic endothelial cells (MEECs) lined the lateral channels to form *in vitro* microvessel analogues within a microtissue system.

### Apparent vascular permeability

Collagen hydrogel was added to the central ECM compartment of the device at a concentration of 6 mg ml^−1^ to minimize spontaneous sprouting. Previously, we confirmed using confocal microscopy that in the microtissue system endothelial cells form a confluent monolayer at the endothelial cell/ECM interface (Chang et al., 2020). MEECs were cultured in the microtissue system for 3 days prior to permeability measurements: 24 h with control media and the subsequent 48 h with the respective media, conditioned or otherwise. Endothelial media was added to T-75 culture flasks with CAFs at approximately 90% confluency and incubated for 24 h to generate conditioned media. Conditioned media was freshly prepared for each experimental run. A volume of 2 µl of 70 kDa Texas-Red-conjugated Dextran was passively loaded into one of the MEEC-lined microchannels as a fluorescent tracer at a concentration of 0.05 mM. Dye diffusion across the interface of the vessel microchannel into the collagen ECM channel was detected using time-lapse epifluorescent microscopy (TS-100, Nikon) over the course of 3 hours under temperature-controlled incubator conditions. Changes in fluorescence intensity within the adjacent ECM region were quantified using NIH ImageJ software to define apparent vascular permeability. Apparent permeability, $P_{App}$ , was calculated using Eq. 1:

$$P_{App}=\left(\frac{1}{\Delta I}\right)\times \left(\frac{dI}{dt}\right)\times \left(\frac{V_{v}}{S_{v}}\right)$$ (1)

where ΔI is the initial source intensity in the vessel channel immediately proximate to each aperture, dI/dt is the change in intensity over time in the adjacent ECM region, and $V_{v}/S_{v}$ is the volume-to-surface area ratio of the dye-loading microvessel analogue channel (Chang et al., 2020).

### Collagen gel contraction assay

The contractility of fibroblasts was measured to quantify the physical remodeling of fibroblast-laden collagen gels (Ren et al., 2019; Zhang et al., 2019). Collagen gels were prepared by adding fibroblasts suspended in cell media at a density of 1.0 × 10^6^ cells/ml to a type I collagen solution (Corning) at a concentration of 1 mg/ml. A total volume of 500 µl was cast into each well of a 24-well plate with four replicates per condition. Collagen gels polymerized for 30 min at 37°C and 5% CO_2_ in a humidified incubator. Following polymerization, an additional 500 µl of fibroblast media was added to each well. AMD3100 (Tocris) was reconstituted to a concentration of 10 µM in fibroblast media to passively diffuse into collagen hydrogels for experimental conditions involving the CXCR4-blocking agent. Images of gels were taken with a stereo microscope (Nikon, SMZ18) across 3 days. The surface area of the gels was quantified by NIH ImageJ software at each time point. The percent contraction was determined by Eq. 2:

$$Contraction\;percentage\;\left(\%\right)=\frac{A_{well}-A_{gel}}{A_{well}}\times 100$$ (2)

where $A_{well}$ and $A_{gel}$ are the surface area of the well and fibroblast-embedded collagen gel, respectively.

### Statistical analysis

Each experimental condition was conducted at least in triplicate. Reported values represent the mean ± standard deviation. Ordinary one-way analysis of variance (ANOVA) was carried out for apparent permeability data with Dunnett’s multiple comparisons test to identify statistical significance compared to control. Two-way ANOVA was performed for analysis of contraction assay with Tukey’s multiple comparisons test to compare between groups. Post hoc unpaired, two-tailed Student *t*-tests were performed, as necessary. Data were analyzed using GraphPad Prism 9 (GraphPad Software), and statistical significance was set to α = 0.05: *p ≤ 0.05, **p ≤ 0.01, ***p < 0.001.

## Results and discussion

### Subclasses of cancer-associated fibroblasts and CXCR4 expression

**FIGURE 1:**
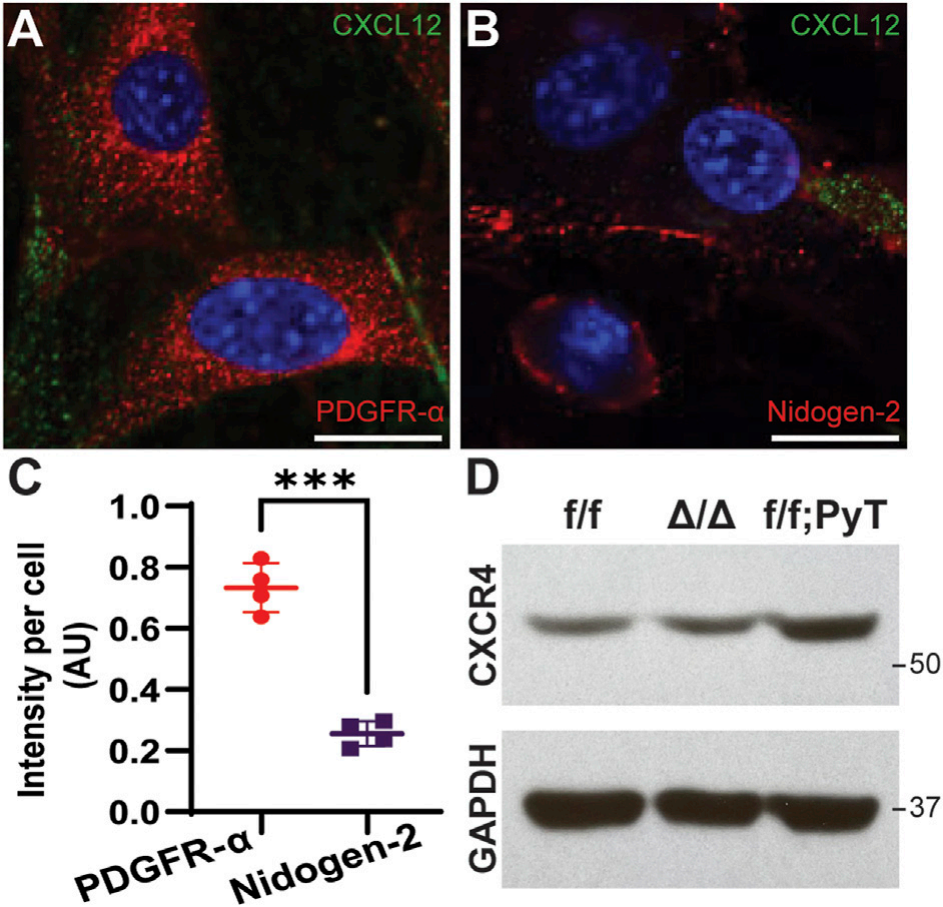
Molecular characterization of fibroblasts isolated from mouse mammary glands. **(A**,**B)** Representative confocal images of cancer-associated fibroblasts (CAFs) by immunofluorescence (IF) staining of CXCL12 (green) and PDGFR-α (red for **A**) and Nidogen-2 (red for **B**). Nuclei (blue) were counterstained with DAPI. Scale bars are 20 µm. **(C)** Quantification of fluorescence intensity of PDGFR-α and Nidogen-2 per cell from confocal IF images. Values represent the mean ± standard deviation, with *n* = 4 technical replicates: *p ≤ 0.05, **p ≤ 0.01, ***p < 0.001 from unpaired *t*-test. **(D)** Western blot analysis of CXCR4 from fibroblast lysates derived from mouse mammary glands. f/f: floxed control allele MMFs, ∆/∆: CXCL12-knockout MMFs, f/f; PyT: CAFs from tumor-bearing mammary glands of Cxcl12^f/f^;PyT mice. CXCR4 expression is preserved in the CXCL12 genetically ablated fibroblasts and robust in CAFs.

Subpopulations of breast cancer-associated fibroblasts have been previously reported by interrogating the transcriptome of isolated murine CAFs using single-cell RNA-sequencing (scRNA-seq) (Bartoschek et al., 2018). The proposed taxonomy of CAFs in breast cancer defined discrete subtypes by their gene expression profiles. Among the subclasses, vascular CAFs (vCAFs) and matrix CAFs (mCAFs) play important roles in breast tumorigenesis and were of particular interest given the context of our microfluidic model, which is constituted by matrix-embedded CAFs in proximity to microvessel analogues. To better characterize our f/f; PyT CAFs at the molecular level, we performed immunofluorescence (IF) on isolated cells to stain for the vCAF marker Nidogen-2, and the mCAF marker PDGFR-α (Figures 1A,B). We found both Nidogen-2-positive vCAFs and PDGFR-α-positive mCAFs; however, the fluorescence intensity of PDGFR-α was nearly 3-fold higher than that of Nidogen-2, suggesting a greater subpopulation of mCAFs than vCAFs (Figure 1C). Furthermore, each subclass of CAFs is attributable to a putative origin (Bartoschek et al., 2018): vCAFs derive from perivascular cells that invade the tumor stroma, and mCAFs originate from co-opted resident fibroblasts. Thus, we conclude that we had a greater preponderance of mCAFs originating from local fibroblasts that had been co-opted by the breast tumor. The higher PDGFR-α signal exhibited by our f/f; PyT CAFs harvested from 8-week-old mice is also in agreement with Bartoschek et al. (2018) who reported that PDGFR-α-positive mCAFs from the MMTV-PyMT tumor mouse model were most prevalent at 8 weeks and decreased during tumor progression through 15 weeks.

We next examined the relative basal levels of CXCR4 protein expression among the fibroblasts *via* Western blot analysis. Floxed control allele MMFs (f/f), CXCL12-knockout MMFs (∆/∆), and CAFs from tumor-bearing Cxcl12^f/f^;PyT mice (f/f; PyT) were isolated from mouse mammary glands and included for analysis. The fibroblast-specific gene deletion of CXCL12 did not have a significant effect on CXCR4 expression evidenced by comparing ∆/∆ fibroblasts to their wild-type counterparts (f/f) (Figure 1D). Moreover, the receptor CXCR4 is robustly expressed in f/f; PyT CAFs and qualitatively higher compared to MMFs. Taken together, we conclude that CXCR4 expression is preserved in the CXCL12 genetically ablated fibroblasts, and that disruption of CXCL12 signaling *via* competition with AMD3100 to CXCR4 is possible in each of the fibroblast cell lines.

### Fibroblast-derived CXCL12 is a major driver of vessel permeability

**FIGURE 2:**
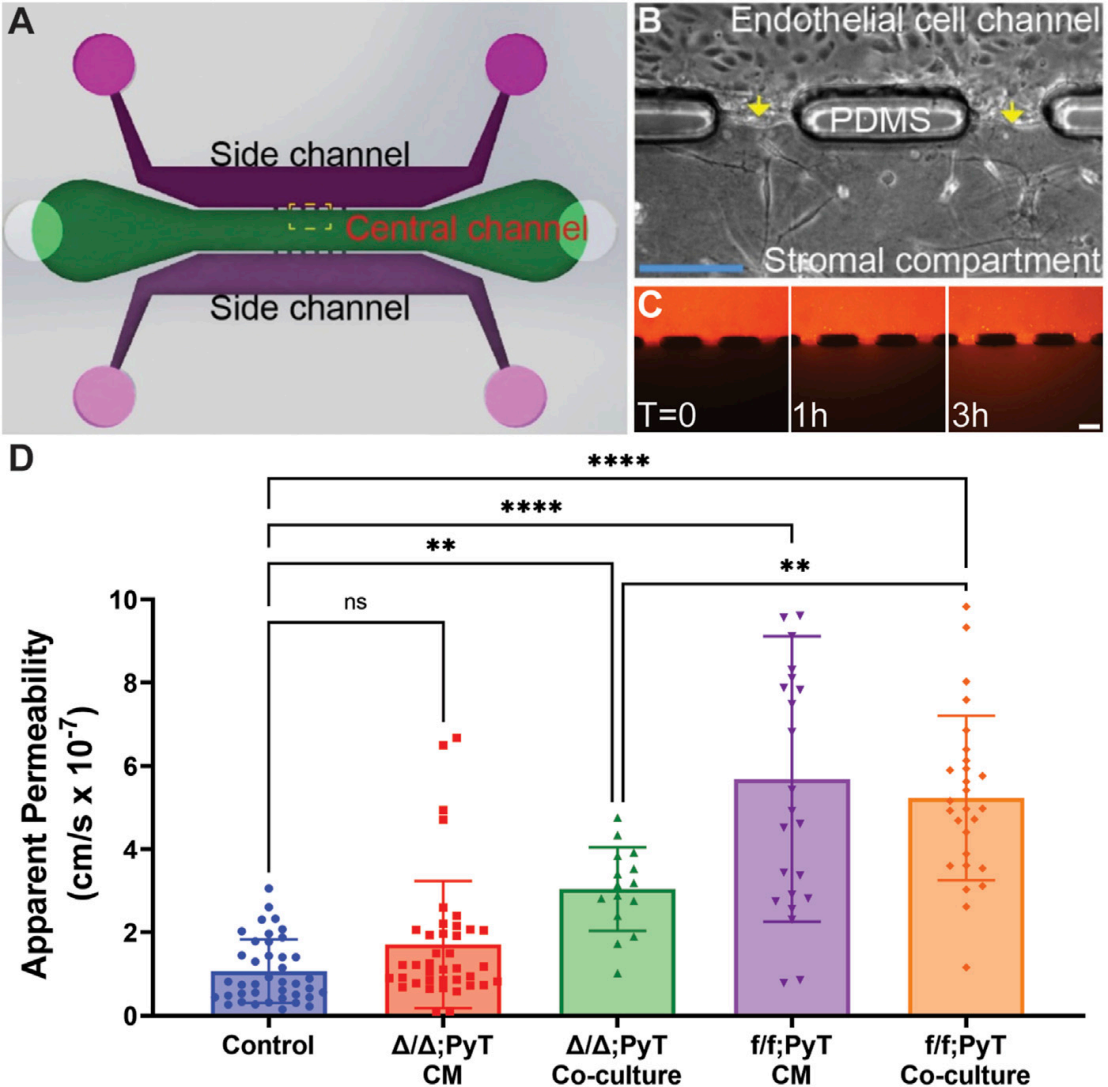
Compartmentalized microfluidic device for vascular permeability measurements. **(A)** Schematic of microtissue analogue microfluidic device. **(B)** Dashed boxed region in **(A)** depicting endothelial cell-lined microchannel laterally adjacent to a central stromal compartment containing fibroblasts embedded in a 3-D type I collagen gel. Arrows depict the endothelial-stromal compartment interface. **(C)** Texas Red-conjugated Dextran (70 kDa) dissolved in media served as a fluorescent tracer and was introduced into the MEEC-lined channel. The fluorescent tracer passes through the endothelial monolayer of the microvessel analogue and enters the stromal compartment by diffusion. Scale bars are 50 µm. **(D)** Changes in fluorescence intensity within the stromal compartment were used to calculate apparent vascular permeability of vessels in the presence of conditioned media (CM) or co-cultured with CAFs—with and without Cxcl12 gene deletion. Reported values represent the mean ± standard deviation, with *n* ≥ 3 biological replicates and at least 15 apertures analyzed for each condition: *p ≤ 0.05, **p ≤ 0.01, ***p < 0.001.

Our microtissue analogue device was employed to investigate the role of soluble fibroblast-derived CXCL12 on endothelial vessel function (Chang et al., 2020). MEECs lined one microchannel of the device, which laterally interfaced with an adjacent stromal compartment containing a 3-D hydrogel of type I collagen (Figures 2A,B). Texas Red-conjugated Dextran (MW ∼70 kDa) dissolved in media served as a fluorescent tracer. Changes in fluorescence intensity within the stromal compartment were used to calculate apparent vascular permeability (Figure 2C). These measurements were obtained for MEEC microvessel analogues in the presence of conditioned media (CM) collected from respective CAFs or from co-culture models in which CAFs were embedded within the collagen gel matrix in the central compartment. As a control condition, the apparent permeability of MEEC-lined channels was measured using normal endothelial culture media in the absence of fibroblasts.

The presence of the chemokine CXCL12 from f/f; PyT CAFs significantly increased the vascular permeability of endothelial microvessel analogues approximately 5-fold in both conditioned media and the co-culture model: means of 5.7 and 5.2 × 10^–7^ cm/s, respectively, compared to the control arm of 1.1 × 10^–7^ cm/s (Figure 2D). Given that the values from CM and co-culture models were not significantly different from each other, the presence of CAFs and soluble CXCL12 did not appear to have an additive effect on permeability. In the absence of CXCL12 from CAFs with the fibroblast-specific CXCL12 deletion (Δ/Δ; PyT), conditioned media did not have a significant effect on vessel permeability compared to control. Notably, conditioned media from CAFs contains a vast myriad of growth factors, cytokines, and other extracellular matrix protein constituents of the CAF secretome. Therefore, the principal difference between Δ/Δ; PyT conditioned media and f/f; PyT conditioned media is the absence of fibroblast-derived CXCL12. Consequently, soluble CXCL12 can be attributed as a primary driver of this observed rise in vascular permeability operating through a paracrine signaling axis between CAFs and nearby endothelia.

**FIGURE 3:**
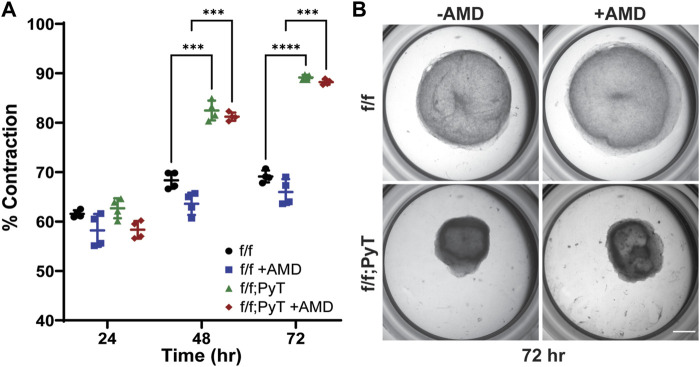
Collagen gel contraction assay to determine fibroblast contractility. **(A)** Percent contraction of normal mouse mammary gland fibroblasts (f/f) and CAFs (f/f; PyT) harvested from PyT tumor-induced mice, with and without the pharmacological CXCR4 inhibitor, AMD3100 (AMD). CAFs exhibit greater contractility compared to their normal counterparts by 48 h. AMD did not have a statistically significant effect on fibroblast contraction, although a slight reduction in mean contraction is observed for both cell lines. Reported values represent the mean ± standard deviation, with *n* = 4 technical replicates for each condition: *p ≤ 0.05, **p ≤ 0.01, ***p < 0.001. **(B)** Representative images of fibroblast-laden collagen gels under stereo microscope after 72 h of contraction. Scale bar is 2 mm.

Interestingly, apparent permeability significantly increased approximately 3-fold in the CXCL12-depleted CAF co-culture model (Δ/Δ; PyT), with a mean of 3.0 × 10^–7^ cm/s, compared to control. This suggests that despite the absence of soluble CXCL12, CAFs are capable of augmenting vessel permeability within the local tumor microenvironment through other means, for example, indirectly through ECM remodeling or TME reprogramming. Of note, CAFs are known to produce excess collagen leading to increased matrix density, desmoplasia, and tissue stiffness (Mouw et al., 2014; Shen et al., 2020). A highly fibrotic tumor can also cause immunosuppression by excluding T lymphocytes through various mechanisms, and thus promotes tumor survival (Chen et al., 2019). In addition, tissue stiffness has been shown to promote a leakier tumor-like vasculature and angiogenesis (Bordeleau et al., 2017), thereby suggesting an interplay between fibroblast contractility, matrix stiffness, and an altered vascular phenotype. These findings prompted us to investigate the autocrine signaling effect of CXCL12 on fibroblasts’ ability to remodel the ECM. Namely, the contractility of fibroblasts—both CAFs and mouse mammary fibroblasts (MMF) from normal glands—on type I collagen was investigated (Figure 3).

### CXCL12-mediated fibroblast remodeling of the ECM

*In vitro* collagen gel contraction assays were performed for MMFs and CAFs harvested from their respective mice models in the presence and absence of AMD3100 (AMD). AMD3100, a pharmaceutical also known as plerixafor, is a highly selective antagonist to the CXCR4 receptor, thus, it directly competes with endogenous CXCL12. The pharmacological blocking of soluble CXCL12 was used to disrupt the autocrine signaling axis of CXCL12 secreted by both normal fibroblasts and CAFs. Collagen hydrogels contracted more than 55% after 24 h for both CAF and MMF conditions confirming the ability of these fibroblasts to contract ECM *in vitro* (Figure 3A). While contractility increased for all four conditions over 3 days, CAFs exhibited significantly greater contractility compared to their normal counterparts by Day 2 lasting through the duration of the assay (Figure 3B). By Day 3, mean contraction for CAF-laden gels had reached 88 and 89%, with and without AMD, respectively. By contrast, normal mammary fibroblasts contracted 66 and 69%, with and without AMD, respectively. Overall, AMD3100 did not have a statistically significant effect on fibroblast contraction, although, a modest drop in mean contraction was observed for both cell lines. As a result, the antagonistic blocking of autocrine CXCL12 by AMD3100 does not significantly affect fibroblasts’ ability to remodel the local ECM. The primary upstream trigger of CAF contractility is likely not CXCL12, such that autocrine effects of CXCL12 are probably not responsible for making fibroblasts substantially more contractile. Taken together, CXCL12 from CAFs increases the permeability of surrounding microvessel analogues—which has implications for tumor intravasation and metastasis—however, CXCL12 does not seem to play a major role in the ECM remodeling of CAFs by autocrine control.

## Conclusion

Stromal CXCL12 secreted by CAFs has a potent effect on increasing the vascular permeability of local blood microvessels through paracrine signaling of the chemokine. A leakier endothelium is of particular importance given its role in angiogenesis and intravasation by tumor cells within the broader context of the metastatic cascade. Furthermore, co-culture with CAFs that had a CXCL12-specific genetic ablation resulted in a reduction of vascular permeability compared to CXCL12-secreting CAFs—presumably through indirect matrix remodeling. Despite CAF-secreted CXCL12 promoting tumor-like vasculature, CXCL12 does not appear to control fibroblast contractility through upstream autocrine signaling mechanisms.

## Data Availability

The raw data supporting the conclusion of this article will be made available by the authors, without undue reservation.
